# What’s new on the treatment of pedophilia and hebephilia?

**DOI:** 10.1192/j.eurpsy.2023.2333

**Published:** 2023-07-19

**Authors:** J. Castro Rodrigues, M. Vieira, B. F. da Silva, L. M. Ribeiro

**Affiliations:** 1 Psychiatry, Hospital Center of Vila Nova de Gaia/ Espinho, Vila Nova de Gaia, Portugal

## Abstract

**Introduction:**

Paraphilias constitute a set of psychiatric conditions that are often chronic and require a combination of treatment approaches, such as pharmacotherapy and psychotherapy. Sexual interest toward prepubescents and pubescents (pedophilia and hebephilia) is frequently identified in criminal settings, within numerous child sexual abuse and child pornography offenses. The high prevalence rates and negative consequences of these acts, causing distress in multiple important areas of health and functioning, reveal the importance of preventing these offenses as a clinical and social matter. Secondary prevention programs, which provide treatment and support for those with paraphilia disorders before sexually abusive behaviors and legal system involvement, show as ethically and socially necessary.

**Objectives:**

We aim to discuss and bring insights into the knowledge on pedophilia and hebephilia treatments and prevention programs, in the fields of psychotherapy as well as pharmacologic strategies.

**Methods:**

We present a non-systematic review of the updated literature on this subject from the data found on the *PubMed* and *PsycInfo* databases.

**Results:**

Preliminary results of recent works show that at-risk individuals with paraphilia disorders are often willing to seek treatment without external pressure from the legal system, and report benefits from early treatments. Most studies found that gonadotropin-releasing hormone agonists reduce the risk of child sexual abuse in men with pedophilia. An injectable form has shown to lower this risk 2 weeks after the initial injection, suggesting its use as a rapid-onset treatment option. Cyproterone acetate and medroxyprogesterone acetate are other anti-androgen drugs that inhibit hypersexual behavior, with important side effects to be considered. The combination of androgen deprivation treatment and psychotherapy has a greater effect on preventing fantasies, urges, and behaviours in paraphilic patients. Cognitive-behavioural psychotherapy shows the best results and should soon be initiated in all patients. Biomolecular studies revealed that serotonin and prolactin inhibit sexual arousal, being SSRIs used as first treatment in younger patients, particularly in less severe cases.

**Conclusions:**

Evidence-based treatments from randomized clinical trials for paedophilic and hebephilic disorders are lacking. These current numbers reveal the need for widespread implementation of primary and secondary prevention initiatives, that go beyond the prevention of a repeated offense. There is a need for further research using controlled, randomized trials to examine the effectiveness of sexual offender treatment including psychotherapeutic and pharmacologic interventions. The development of more specific, more effective, and better-tolerated medications for these disorders should be recognized as a program worthy of greater support from government and pharmaceutical industry sources.

**Disclosure of Interest:**

None Declared

